# *Acanthopanax trifoliatus* (L.) Merr polysaccharides ameliorates hyperglycemia by regulating hepatic glycogen metabolism in type 2 diabetic mice

**DOI:** 10.3389/fnut.2023.1111287

**Published:** 2023-02-09

**Authors:** Yuzi Lin, Jinghua Pan, Yue Liu, Huiwen Yang, Guoyu Wu, Yufang Pan

**Affiliations:** ^1^School of Pharmacy, Guangdong Pharmaceutical University, Guangzhou, China; ^2^Key Specialty of Clinical Pharmacy, The First Affiliated Hospital of Guangdong Pharmaceutical University, Guangzhou, China; ^3^Guangdong Provincial Key Laboratory of Advanced Drug Delivery and Guangdong Provincial Engineering Center of Topical Precise Drug Delivery System, Guangdong Pharmaceutical University, Guangzhou, China

**Keywords:** *Acanthopanax trifoliatus* (L.) Merr, type 2 diabetes mellitus, PI3K/Akt/GSK3β, cAMP/PKA, AMPK, liver glucose metabolism

## Abstract

**Introduction:**

Drug monotherapy was inadequate in controlling blood glucose levels and other comorbidities. An agent that selectively tunes multiple targets was regarded as a new therapeutic strategy for type 2 diabetes. *Acanthopanax trifoliatus* (L.) Merr polysaccharide (ATMP) is a bio-macromolecule isolated from *Acanthopanax trifoliatus* (L.) Merr and has therapeutic potential for diabetes management due to its anti-hyperglycemia activity.

**Methods:**

Type 2 diabetes mellitus was induced in mice using streptozotocin, and 40 and 80 mg/kg ATMP was administered daily via the intragastric route for 8 weeks. Food intake, water intake, and body weight were recorded. The fasting blood glucose (FBG), fasting insulin (FINS) and an oral glucose tolerance test (OGTT) were performed. Histological changes in the liver and pancreas were analyzed by H&E staining. The mRNA and the protein levels of key factors involved in glycogen synthesis, glycogenolysis, and gluconeogenesis were measured by quantitative real time PCR and Western blotting.

**Results:**

In this study, we found that ATMP could effectively improve glucose tolerance and alleviate insulin resistance by promoting insulin secretion and inhibiting glucagon secretion. In addition, ATMP decreases glycogen synthesis by inhibiting PI3K/Akt/GSK3β signaling, reduces glycogenolysis *via* suppressing cAMP/PKA signaling, and suppresses liver gluconeogenesis by activating AMPK signaling.

**Conclusion:**

Together, ATMP has the potential to be developed as a new multitargets therapeutics for type 2 diabetes.

## 1. Introduction

Type 2 diabetes mellitus (T2DM) is a complex chronic metabolic disorder resulting from a deficiency of insulin secretion and insulin resistance (1). In patients with T2DM, chronically elevated blood glucose levels can cause multiple organ dysfunctions and a series of complications (2). Weight loss *via* lifestyle changes, which can partly suppress hyperglycemia, is the primary strategy to manage T2DM (3). Pharmacological treatment is necessary when glycemic control is not achieved after lifestyle intervention. Chemical drugs, such as acarbose, glibenclamide, and rosiglitazone, have been widely used to treat T2DM. However, they are associated with several undesirable side effects, including hypoglycemia, fluid retention, osteoporosis, and heart failure (2). These limitations have raised substantial concerns. Therefore, it is critical to develop drugs that have fewer side effects without loss of insulin sensitization for improved therapies for T2DM.

Recently, bioactive compounds isolated from natural plants have been noticed because of their low toxicity and high efficacy in therapeutic use for the prevention and treatment of T2DM (1). Polysaccharides are natural macromolecular polymers that are predominantly found in various parts of plants, microorganisms, algae and animals. Polysaccharides have a variety of biological activities such as anti-inflammatory, antioxidant, anti-tumor and anti-diabetic activities, making them one of the most promising candidates in biomedical and pharmaceutical fields. (4, 5). For instance, *Sargassum fusiforme* polysaccharide (6), *Pseudostellaria heterophylla* polysaccharide (7) and *Okra* polysaccharide (8), have been proposed as alternative medicines used in diabetic treatment. *Acanthopanax trifoliatus* (L.) Merr is a drug food homologous plant, which belongs to non-toxic substance. It was proved that *Acanthopanax trifoliatus* (L.) Merr has no genotoxicity and has no significant toxic effect on blood routine, blood biochemistry and internal organs (9, 10). *Acanthopanax trifoliatus* (L.) Merr polysaccharides (ATMP), a bio-macromolecule isolated from the dried rhizome of *Acanthopanax trifoliatus* (L.) Merr, has been reported various pharmacological properties such as antioxidant (11), anti-inflammatory (12), and anticancer (13). Previous studies showed that ATMP has a therapeutic effect on T1DM by regulating the balance of CD4^+^/CD8^+^ cells and Th1/Th2 *via* the PPAR-γ signaling pathway (14). And ATMP could inhibit the activity of intestinal disaccharidase and down-regulate intestinal sugar absorption in type 2 diabetic mice (15), suggesting that ATMP might be a candidate for the treatment of T2DM. The molecular mechanism of the use of ATMP for T2DM treatment remains to be fully explored.

Glucose metabolism plays a central role in the pathogenesis of T2DM (16). Multiple processes are involved, including glycogenesis, glycogenolysis, gluconeogenesis and glycolysis. Insulin and glucagon work together in a balance to keep blood glucose levels in a normal range (17). PI3K/Akt/GSK3β signaling pathway plays an important role in glycogenesis. Glycogen synthesis kinase-3β (GSK3β) and glycogen synthesis (GS) are two key enzymes in liver glycogenesis (18). Insulin can activate phosphatidylinositol-3-kinase (PI3K), then activate PI3K phosphorylates protein kinase B (Akt) and act on a series of downstream molecules such as GSK3β (19, 20). Activation of GSK3β promotes the phosphorylation of GS and inhibits its activity, then suppresses glycogen synthesis (21, 22). Glycogen phosphorylase (GP) is the rate-limiting enzyme in glycogenolysis (23). The activation of GP is mediated by cAMP/PKA signaling. An abnormally increased level of active GP was observed in the case of diabetes (24). Gluconeogenesis refers to the process in which non-sugar substances are converted into glucose (25). Excessive gluconeogenesis leads to hyperglycemia. PEPCK and G6Pase are two key enzymes in gluconeogenesis, and the transcriptions of them are regulated by adenosine monophosphate-activated protein kinase (AMPK) (26). Inhibition of PEPCK and G6Pase could reduce gluconeogenesis in the liver, and lower the blood glucose concentration.

Here, we investigated the molecular mechanism of the use of ATMP for T2DM treatment, showing that ATMP could inhibit glycogenesis *via* PI3K/Akt/GSK3β signaling pathway, suppress glycogenolysis by inhibiting cAMP/PKA signaling and reduce gluconeogenesis through activating the AMPK signaling pathway. This study elucidates a novel mechanism of ATMP that ameliorates hyperglycemia by affecting hepatic glycogen metabolism in T2DM mice, providing a theoretical basis for its potential future clinical applications.

## 2. Materials and methods

### 2.1. Preparation of ATMP

*Acanthopanax trifoliatus* (L.) Merr polysaccharide (ATMP) was extracted as previously described with modifications (14). The dry stem of *Acanthopanax trifoliatus* (L.) Merr was obtained from Enping (Guangdong, China). The powdered stem of *Acanthopanax trifoliatus* (L.) Merr was extracted with distilled water 1:10 (*w*/*v*) for 2 h at 100°C. The same process was repeated two times. The solvent was concentrated to 1/10 of the total volume under reduced pressure, and then the supernatants were gathered after centrifugation (4,500 r/min for 10 min). Two times absolute ethanol was added to the supernatant fluid, then the precipitations were collected by centrifuging at 4,500 r/min for 10 min after precipitating for 24 h. To remove water, sticky substances, and other impurities from the material, the precipitation was washed with absolute ethanol, diethyl ether, and acetone in turn, and the *Acanthopanax trifoliatus* (L.) Merr crude polysaccharide (ATMCP) was obtained by vacuum freeze-drying. After removing proteins *via* the Sevag method (27), ATMCP was dissolved in distilled water and centrifuged (8,000 r/min for 10 min). Consequently, the supernatants were purified successively by chromatography on the DEAE-52 cellulose column and then on the SephadexG-75 gel column. The eluent was collected, freeze-dried, and labeled as *Acanthopanax trifoliatus* (L.) Merr polysaccharides (ATMP).

### 2.2. Animal experiments

C57BL/6 mice are sensitive to high-fat feeds. This strain becomes obese, hyperglycemic and insulin resistant when fed a diabetogenic diet, so they were chosen as experimental animals (28). C57BL/6 mice (18.0 ± 2.0 g) were purchased from the Guangdong Medical Laboratory Animal Center and housed in a specific pathogen-free (SPF) animal room under conditions of controlled temperature at 21 ± 1°C and humidity at 60 ± 10%, in a 12 h/12 h light/dark cycle.

After 1 week of acclimatization, the mice were randomly divided into the following groups: NC (normal control, regular diet), and high-fat diet (HFD). The high-fat diet: 58.5% ordinary diet, 15% sucrose, 10% lard fat, 10% protein, 5% milk powder, 1% cholesterol, and 0.5% sodium deoxycholate (Beijing boaigang biological technology company). The mice in the HFD group were fed on a high-fat diet for 4 weeks, then were fasted overnight and drank water freely, and then received an intraperitoneal injection of 130 mg/kg streptozotocin (Sigma, United States). After 7 days/10 days, the fasting blood glucose (FBG) levels of the mice were measured. The mice with an FBG ≥ 16.7 mmol/l were regarded as diabetic.

Thirty diabetic mice were randomly divided into five groups (6 animals/group): normal control group (NC), diabetic control group (DC), metformin group (185 mg/kg/day, Met), and two ATMP treated groups (40 and 80 mg/kg/day, referred as L-ATMP and H-ATMP group, respectively). Mice in Met and ATMP groups were administrated intragastrically once a day, while the mice in the NC and DC groups received the same volume of pure water. The mice in DC, Met, and ATMP groups were fed the high-fat diet and the mice in the NC group were fed a normal diet. The doses of ATMP and administration period in the experiment were determined based on the information from previous studies (14).

The animal experiments were approved by the Ethics Committee of the experimental animal center of Guangdong Pharmaceutical University.

### 2.3. Body weight, food intake, water intake, and fasting blood glucose

Body weight, water intake, and food intake were measured every week. All mice fasted for 6 h, and the blood samples were collected from the tail veins of mice, then the FBG levels were determined using a blood glucose meter (SANNUO, China) once a week.

### 2.4. Determination of oral glucose tolerance test (OGTT)

After 8 weeks of treatment, the OGTT was conducted after 6 h fast. Mice fasted for 6 h and then were orally administered 2 g/kg of glucose (Xiwang, China). The blood glucose levels were measured at 0, 15, 30, 60, 120, and 150 min. The areas under the curve (AUC) of blood glucose levels were calculated to evaluate glucose tolerance.

### 2.5. Measurement of biochemical parameters

After the 8 weeks of treatment, mice fasted for 12 h. The blood samples were obtained from the orbital sinus and centrifuged (4,000 rpm for 15 min at 4°C) to isolate the serum, which was then stored at −80°C until analysis. The mice were then sacrificed by cervical dislocation. The liver tissues were harvested, weighed, and separated into two specimens. One sample was immediately frozen at −80°C, and the other was fixed in 4% paraformaldehyde solution for histopathological analysis. The glycogen contents in the liver were determined by colorimetric assay using biochemical kits (Nanjing Jian Cheng Bioengineering Institute, China). The fasting serum glucagon and fasting insulin (FINS) were assayed using ELISA kits (Quanzhou Ruixin Biotechnology Co., Ltd., China) according to the manufacturer’s instructions. Homeostasis model assessment of insulin resistance (HOMA-IR) was calculated according to the following formulas: HOMA-IR = FBG (mmol/L) × FINS (mIU/L) /22.5.

### 2.6. Histopathological analysis

The fresh liver and pancreas tissues were fixed in a 4% paraformaldehyde solution for 24 h and embedded in paraffin. The liver tissues were then cut into 4 μm slices for periodic acid-Schiff (PAS) staining and hematoxylin–eosin (H&E) staining. Pancreatic tissues were cut into 4um for hematoxylin–eosin (H&E) staining. Photomicrographs were taken using a light microscope (Nikon Eclipse E 100, Tokyo, Japan).

### 2.7. RNA extraction, reverse transcription, and real time-PCR

Total RNA was isolated from liver tissues using TRIzol reagent (Accurate Biology, Hunan, China). Subsequently, RNA samples were reverse transcribed into cDNA using the Evo M-MLV RT Kit (Accurate Biology, Hunan, China). The cDNA was used as the template for the real-time quantitative PCR (RT-qPCR) reaction. The primers were synthesized by Sangon (Shanghai, China). RT-qPCR was performed using SYBR^®^ Green Premix Pro Taq HS qPCR Kit (Accurate Biology, Hunan, China) according to the manufacturer’s instructions. RT-qPCR is carried out in the C1000Touch^™^ Thermal cycle System (Bio-Rad, CA, United States). The relative expression for a particular gene was calculated using the method of 2^–ΔΔC^. The primer sequences were listed in Table 1.

**Table 1:** Primer used for RT-qPCR experiments.

Gene	Sequence (5′–3′)
G6Pase-F	TCAGCCACATCCACAGCATCTATAATG
G6Pase-R	CCAGAGTCCACAGGAGGTCTACAC
Foxo1-F	ACATCTGCCATGAACCGCTTGAC
Foxo1-R	CACCCATCCTACCATAGCCATTGC
IRS1-F	CCAGCAGCAGTAGCAGCATCAG
IRS1-R	GCTTACCGCCACCACTCTCAAC
PI3K-F	GGAATGTCGGGAGCAGCAACC
PI3K-R	TCTACCACTACGGAGCAGGCATAG
GSK3β-F	GACAGTGGTGTGGATCAGTTGGTG
GSK3β-R	TCCTGCTCCTGGTGAGTCCTTTG
GBE-F	CGAACATAAGATGGTGGTTGGAGGAG
GBE-R	TCCGTGGTGATGATAGAGCATAGAGG
AKT-F	TCAGGATGTGGATCAGCGAGAGTC
AKT-R	AGGCAGCGGATGATAAAGGTGTTG
PKA-F	GGTTACAATAAGGCGGTGGACTGG
PKA-R	GGTCAGCAAAGAATGGAGGGTAGC
GP-F	TGAGAGTAGATGATGTGGCTGCTTTG
GP-R	TGTCTTTGAAGAGGTCTGGCTGATTG
Glucagon-F	TAGCAAAGATCCACTCGGACA
Glucagon-R	AGAAGCTGCCTTTTATACTCGT

### 2.8. Western blot analysis

Total proteins of liver tissues were extracted using RIPA lysis buffer (Leagene Biotechnology, China). Protein concentrations were measured using bicinchoninic acid (BCA) protein assay kit (Thermo Fisher Scientific, United States). Samples were analyzed by western blotting as previously described (29). Samples were loaded onto 8 or 10% SDS polyacrylamide gels (Beyotime Biotechnology, China), and then transferred to PVDF membranes (IPVH00010, Millipore, Germany) and immunoblotted using standard techniques Membranes were incubated with freshly mixed ECL solution (Biosharp, China) and visualized with an Automated Imaging System (Tanon, China). Antibodies used for western blotting were β-actin (Cell Signaling Technology, United States), PEPCK (Cell Signaling Technology, United States), PGC-1α (Cell Signaling Technology, United States), Akt (Cell Signaling Technology, United States), p-Akt (Cell Signaling Technology, United States), PKAα (Cell Signaling Technology, United States), p-PKA (Cell Signaling Technology, United States), GS (Abcam, United Kingdom), p-GS (Abcam, United Kingdom), PK (Abcam, United Kingdom), p-PK (Abcam, United Kingdom), AMPK (Abcam, United Kingdom), p-AMPK (Abcam, United Kingdom), FOXO1 (Bioworld, China), p-FOXO1 (Bioworld, China) and GPa (Bioworld, China).

### 2.9. Statistical analysis

Data were analyzed and statistics were performed in SPSS 23.0 (Chicago, IL, United States). Data are presented as mean ± standard deviation (SD). Statistical significance was calculated using Dunnett’s T-test and Least Significant Difference test. Significant differences between the two groups were noted by asterisks (^*^p < 0.05; ^**^p < 0.01).

## 3. Results

### 3.1. *Acanthopanax trifoliatus* (L.) Merr polysaccharide ameliorates the symptoms of T2DM in mice

**Figure 1:**
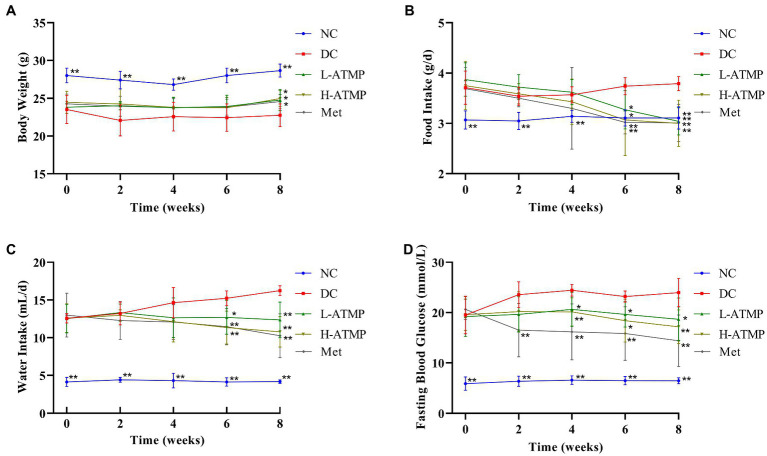
*Acanthopanax trifoliatus* (L.) Merr polysaccharide (ATMP) ameliorates the symptoms of type 2 diabetes mellitus in mice. Body weight **(A)**, food intake **(B)**, water intake **(C)** and fasting blood glucose **(D)** in Type 2 diabetes mellitus (T2DM) mice measured at 0, 2, 4, 6, and 8 weeks after ATMP treatment. Results are expressed as the mean ± SD (*n* = 6). ^*^p < 0.05 and ^**^p < 0.01 vs. DC. NC: normal group; DC: diabetic control group; Met: diabetic mice treated with 185 mg/kg metformin; L-ATMP and H-ATMP: diabetic mice treated with 40 or 80 mg/kg ATMP, respectively.

To examine the anti-diabetic effects of ATMP, the body weight, food intake, water intake, and fasting blood glucose (FBG) changes of the diabetic mice after ATMP treatment were measured. Metformin, which was considered to be the optimal initial therapeutic drug for patients with type 2 diabetes mellitus, was used as a positive control in the experiments (30). The results were shown in Table 2. Compared to untreated diabetic mice, we found that the body weights of the diabetic mice markedly increased after 8 weeks of ATMP treatment (Figure 1A). The food and water intake of the mice in ATMP groups was decreased (Figures 1B,C). The fasting blood glucose levels were measured. Similar to the Met group, the blood glucose levels of mice in ATMP groups were significantly decreased (Figure 1D). These results indicated that ATMP can alleviate the symptoms of T2DM.

**Table 2:** Influence of *Acanthopanax trifoliatus* (L.) Merr polysaccharide (ATMP) on body weight, food intake, water intake and fasting blood glucose.

Items	Group	0 week	2 weeks	4 weeks	6 weeks	8 weeks
Body weight (g)	NC	28.02 ± 0.96^**^	27.43 ± 1.18^**^	26.82 ± 0.75^**^	28.02 ± 0.95^**^	28.68 ± 0.86^**^
	DC	23.52 ± 1.87	22.08 ± 2.05	22.58 ± 1.91	22.45 ± 1.82	22.75 ± 1.50
	L-ATMP	23.82 ± 0.45	24.02 ± 0.54	23.75 ± 1.37	23.92 ± 1.51	24.77 ± 1.40^*^
	H-ATMP	24.48 ± 1.47	24.26 ± 2.01	23.80 ± 1.35	23.75 ± 1.37	24.98 ± 1.05^*^
	Met	24.28 ± 1.29	23.96 ± 1.32	23.77 ± 1.19	23.82 ± 1.05	24.58 ± 0.96^*^
Food intake (g/d)	NC	3.07 ± 0.18^**^	3.05 ± 0.17^**^	3.14 ± 0.12^**^	3.11 ± 0.16^**^	3.11 ± 0.22^**^
	DC	3.71 ± 0.33	3.54 ± 0.18	3.57 ± 0.16	3.74 ± 0.17	3.79 ± 0.14
	L-ATMP	3.87 ± 1.08	3.72 ± 0.25	3.62 ± 0.25	3.27 ± 0.38^*^	3.04 ± 0.27^**^
	H-ATMP	3.75 ± 0.48	3.59 ± 0.20	3.43 ± 0.45	3.07 ± 0.71^*^	3.00 ± 0.46^**^
	Met	3.69 ± 0.42	3.50 ± 0.17	3.30 ± 0.81	3.02 ± 0.23^**^	3.01 ± 0.37^**^
Water intake (mL/d)	NC	4.12 ± 0.60^**^	4.14 ± 0.32^**^	4.31 ± 0.96^**^	4.13 ± 0.55^**^	4.17 ± 0.24^**^
	DC	12.58 ± 0.63	13.21 ± 1.52	14.64 ± 2.01	15.22 ± 0.98	16.24 ± 0.65
	L-ATMP	12.52 ± 1.89	13.32 ± 1.13	12.64 ± 2.63	12.69 ± 2.24^*^	12.36 ± 2.37^**^
	H-ATMP	12.59 ± 1.90	12.95 ± 0.74	12.13 ± 2.71	11.34 ± 2.19^**^	10.77 ± 2.02^**^
	Met	12.99 ± 2.90	12.28 ± 2.52	12.07 ± 2.31	11.46 ± 2.41^**^	10.25 ± 2.91^**^
Fasting blood glucose (mmol/L)	NC	5.87 ± 1.33^**^	6.35 ± 1.01^**^	6.55 ± 0.87^**^	6.47 ± 0.84^**^	6.43 ± 0.58^**^
	DC	19.52 ± 3.73	23.55 ± 2.56	24.43 ± 1.16	23.20 ± 1.11	23.97 ± 2.84
	L-ATMP	19.22 ± 3.97	19.65 ± 3.52	20.65 ± 3.38^*^	19.65 ± 2.54^*^	18.67 ± 4.24^*^
	H-ATMP	19.58 ± 3.13	20.18 ± 3.95	20.15 ± 2.80^**^	18.38 ± 4.27^*^	17.17 ± 3.36^**^
	Met	20.63 ± 2.04	16.52 ± 5.31^*^	16.17 ± 5.55^**^	15.83 ± 5.33^**^	14.38 ± 5.13^**^

Results are expressed as the mean ± SD, (*n* = 6). ^*^p < 0.05 and ^**^p < 0.01 vs. DC. NC: normal group; DC: diabetic control group; Met: diabetic mice treated with 185 mg/kg metformin; L-ATMP and H-ATMP: diabetic mice treated with 40 or 80 mg/kg ATMP, respectively.

### 3.2. *Acanthopanax trifoliatus* (L.) Merr polysaccharide improves the glucose tolerance of diabetic mice

**Figure 2:**
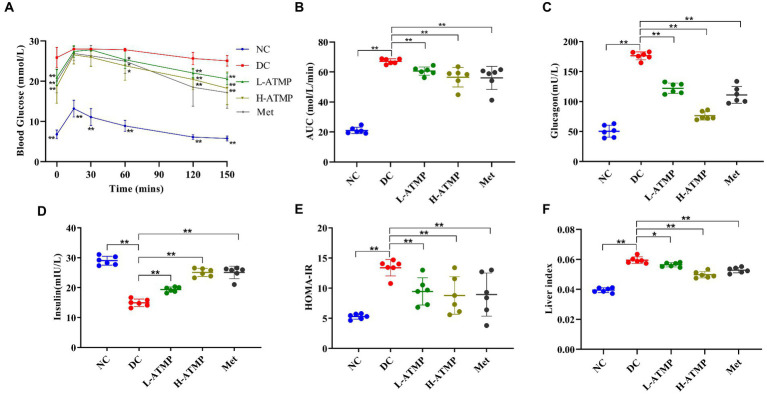
The effect of ATMP in regulating glucose metabolism. The T2DM mice were administered 40 mg/kg or 80 mg/kg ATMP by oral gavage for 8 weeks. **(A)** Oral glucose tolerance tests (OGTT) were performed. Mice were fasted overnight and then orally administered glucose (2.0 g/kg body weight). The blood glucose levels were measured at 0, 15, 30, 60, 120, and 150 min. **(B)** The area under the curve (AUC) of OGTT was calculated using the trapezoidal rule. **(C)** The level of serum glucagon was measured. **(D)** The level of serum insulin was assayed. **(E)** HOMA-IR values were calculated according to the following formulas: HOMA-IR = FBG (mmol/L) × FINS (mIU/L)/22.5. **(F)** The liver index was calculated according to the following formula: Liver Index = liver weight/body weight. Results are expressed as the mean ± SD, (*n* = 6). ^*^p < 0.05 and ^**^p < 0.01 vs. DC. NC: normal group; DC: diabetic control group; Met: diabetic mice treated with 185 mg/kg metformin; L-ATMP and H-ATMP: diabetic mice treated with 40 or 80 mg/kg ATMP, respectively.

To examine the effect of ATMP in regulating glucose metabolism, an oral glucose tolerance test (OGTT) was performed. The glucose tolerance capacity of the diabetic mice was severely damaged. We found that the blood glucose levels in the diabetic mice significantly reduced after 8 weeks of ATMP treatment (Figure 2A). Similar to the Met group, the AUC of mice in ATMP groups notably declined (Figure 2B). These results suggested that ATMP can ameliorate the impaired glucose tolerance in diabetic mice.

### 3.3. *Acanthopanax trifoliatus* (L.) Merr polysaccharide diminishes the serum glucagon content

Glucagon accelerates glycogenolysis and provokes gluconeogenesis in the liver, releasing glucose into the bloodstream (31, 32). Clinical studies show that increased fasting glucagon level is one of the reasons for the elevated glucose level observed in T2DM (33). To elucidate the mechanisms of the hypoglycemic effect of ATMP, the fasting serum glucagon content in diabetic mice after ATMP treatment was determined. We found that the glucagon contents of the mice in ATMP groups were decreased in a dose-dependent way (Figure 2C). High-dose ATMP was superior to metformin in downgrading the level of serum glucagon. These results demonstrated that ATMP could ameliorate hyperglycemia by down-regulating the serum glucagon level in type 2 diabetic mice.

### 3.4. *Acanthopanax trifoliatus* (L.) Merr polysaccharide alleviates insulin resistance

Insulin is an important hormone secreted by islet β cells in response to elevated blood glucose levels (34). To examine the effect of ATMP on insulin in type 2 diabetic mice, the fasting serum insulin level (FINS) was assayed. We found that ATMP dose-dependently increased the FINS of the diabetic mice (Figure 2D). To evaluate the degree of insulin resistance, the homeostasis model assessment of insulin resistance (HOMA-IR) was calculated. Compared to untreated diabetic mice, we found that the HOMA-IR of the diabetic mice was markedly decreased after 8 weeks of ATMP treatment (Figure 2E). The efficiency in improving insulin resistance of ATMP is similar to metformin. These results indicated that ATMP can effectively reduce insulin resistance of T2DM.

### 3.5. *Acanthopanax trifoliatus* (L.) Merr polysaccharide reduces liver glycogen content and ameliorates liver impairment

**Figure 3:**
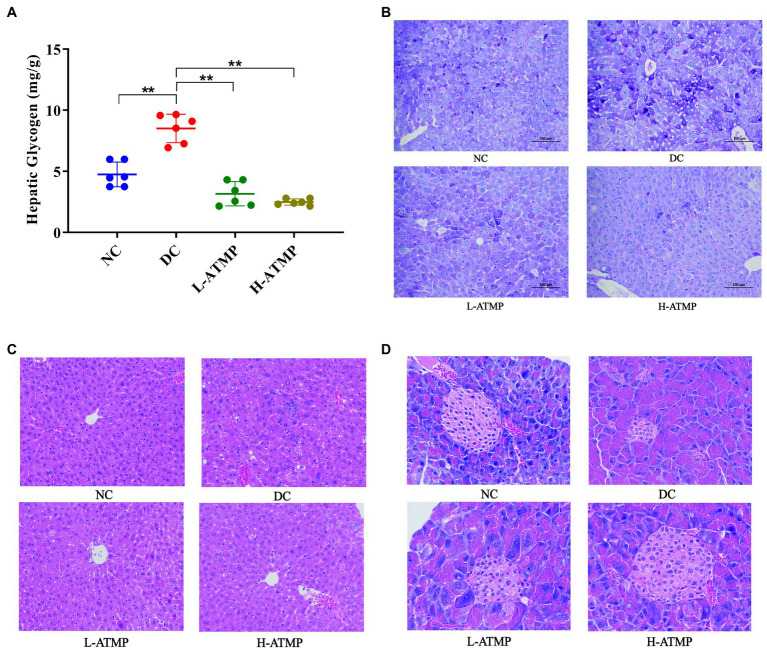
The hepatic glycogen content in T2DM mice decreased after ATMP treatment. The T2DM mice were administered 40 mg/kg or 80 mg/kg ATMP by oral gavage for 8 weeks. **(A)** Liver glycogen contents were determined. **(B)** PAS staining for glycogen content. **(C)** H&E staining of the liver. **(D)** H&E staining of the pancreas. Results are expressed as the mean ± SD, (n = 6). *p < 0.05 and **p < 0.01 vs. DC. NC: normal group; DC: diabetic control group; L-ATMP and H-ATMP: diabetic mice treated with ATMP at 40 or 80 mg/kg, respectively.

Glycogen is the major intracellular storage form of glucose in the liver. Previous studies showed that glycogen level is relevant to insulin resistance in T2DM (35). Excessive accumulation of glycogen in hepatocytes may lead to progressive hepatomegaly and renal enlargement. Next, we investigated the effect of ATMP on regulating liver glycogen content in mice. The liver glycogen content in diabetic mice was markedly higher than that in non-diabetic mice, however, the liver glycogen level dropped substantially after 8 weeks of ATMP treatment (Figure 3A). Periodic acid-Schiff (PAS) staining, which could identify glycogen synthesis, was commonly used to detect the metabolism of liver cells (31). To further investigate the mechanism of ATMP on glycogen metabolism, PAS staining in the liver was performed. In the diabetic mice, glycogens were characterized to be unevenly distributed in the liver parenchyma and lipid vacuoles were found. After ATMP treatment, liver glycogen accumulation and vacuole degeneration decreased (Figure 3B). The results of PAS staining were consistent with that of the liver glycogen content determination. To further explore the effect of ATMP on the histomorphology of liver, H&E staining was performed to evaluate the pathological changes of the tissues. Vacuoles and local inflammatory cell infiltration with loosely arranged hepatocytes were observed in the liver of diabetic mice. ATMP treatment significantly improved hepatic morphology: tighter arranged hepatocytes and fewer vacuoles with reduced volumes. The inflammatory infiltration was also alleviated (Figure 3C). These results revealed that ATMP could reduce liver glycogen content and repair liver impairment in type 2 diabetic mice.

### 3.6. *Acanthopanax trifoliatus* (L.) Merr polysaccharide ameliorates pancreatic impairment in diabetic mice

The pancreas is an important organ for blood sugar control. Impaired pancreatic function contributed to the development of T2DM, thus repairing the function of pancreas was considered a viable treatment for T2DM (36). To investigate the effect of ATMP on the histomorphology of pancreas, H&E staining of the tissues was performed to evaluate the pathological changes. The pancreatic islets of diabetic mice were severely degenerated. An irregular islet structure and decreased islet area were observed in diabetic group. After ATMP treatment, the morphology of pancreatic islets was improved and the shrinkage of the islets was partially prevented (Figure 3D). These results illustrated that ATMP could reduce the degeneration of pancreatic islets in diabetic mice.

### 3.7. *Acanthopanax trifoliatus* (L.) Merr polysaccharide decreases hepatic glycogen synthesis *via* suppressing PI3K/Akt/GSK3β signaling pathway in T2DM mice

**Figure 4:**
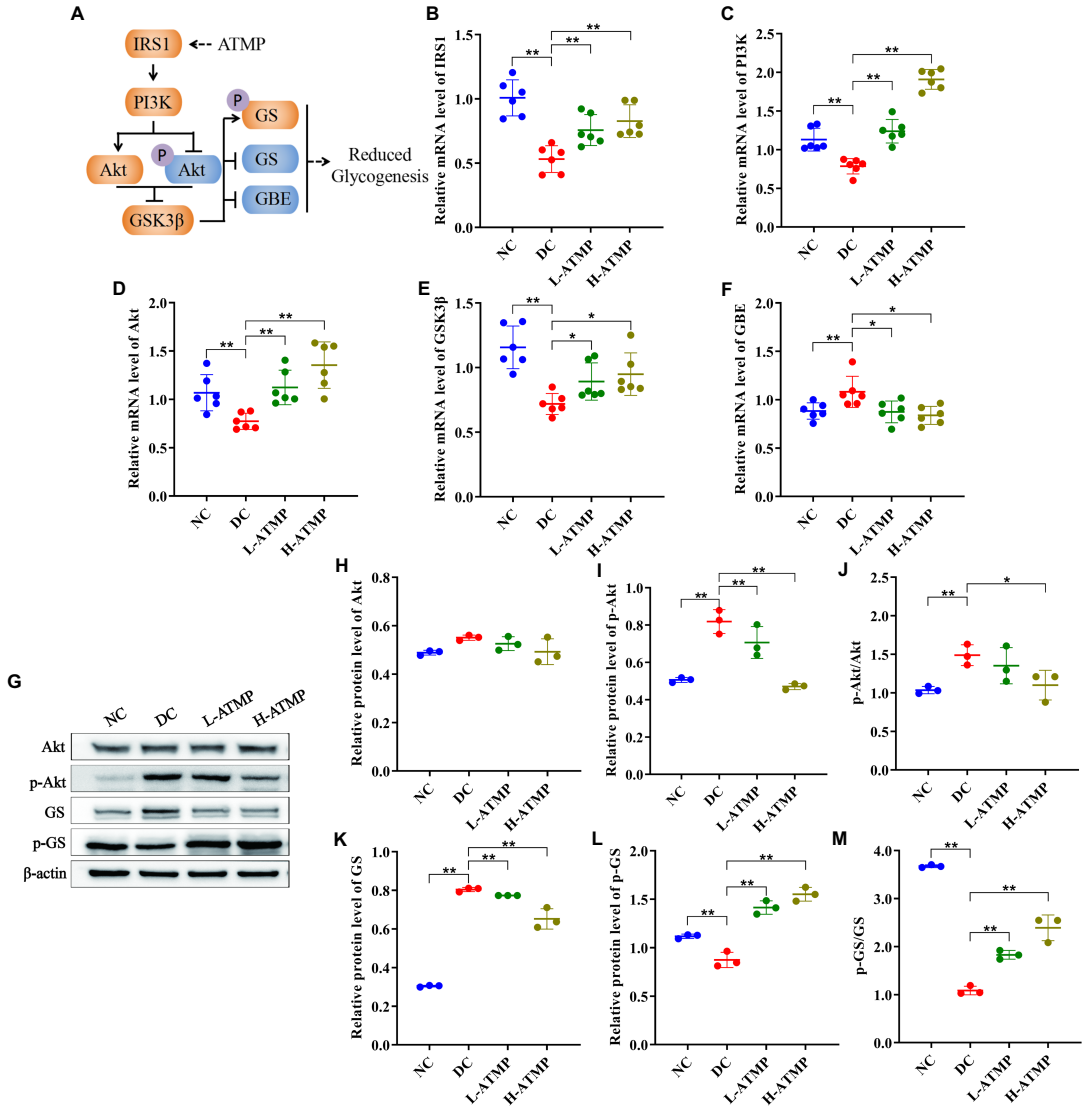
*Acanthopanax trifoliatus* (L.) Merr polysaccharide (ATMP) reduces hepatic glycogen synthesis *via* PI3K/Akt/GSK3β signaling pathway. The T2DM mice were administered 40 mg/kg or 80 mg/kg ATMP by oral gavage for 8 weeks, then the mRNA and protein levels of PI3K/Akt/GSK3β signaling pathway-related proteins were determined. **(A)** Schematic diagram showing ATMP mediating PI3K/Akt/GSK3β signaling to suppress hepatic glycogen synthesis. Orange nodes: up-regulated proteins; Blue nodes: down-regulated proteins. **(B–F)** Relative mRNA levels of IRS1, PI3K, Akt, GSK3β and GBE, and β-actin as a loading control were assayed by RT-qPCR. The relative mRNA levels for proteins of interest were normalized to β-actin. **(G)** Relative protein levels of Akt, p-Akt, GS and p-GS, and β-actin as a loading control were assayed by Western blot. Quantifications of band intensity were normalized to β-actin **(H–M)**. Results are expressed as the mean ± SD. ^*^p < 0.05 and ^**^p < 0.01 vs. DC. NC: normal group; DC: diabetic control group; L-ATMP and H-ATMP: diabetic mice treated with ATMP at 40 or 80 mg/kg, respectively.

PI3K/Akt/GSK3β signaling pathway is known to play an important role in glycogen synthesis (37). To investigate the mechanisms of ATMP to ameliorate the symptoms of T2DM *via* mediating glycogen synthesis, the mRNA level of related molecules involved in the PI3K/Akt/GSK3β signaling pathway was determined by RT-qPCR in all groups. After 8 weeks of ATMP treatment, the mRNA level of IRSI, PI3K, GSK3β, and Akt were elevated significantly while the mRNA expression of GBE was diminished (Figures 4B–F). Furthermore, the protein levels of Akt, p-Akt, GS, and p-GS were evaluated by Western blot. We found that the levels of Akt, p-Akt, p-Akt/Akt, and GS were down-regulated, while the expressions of p-GS and p-GS/GS were dramatically enhanced by ATMP in a dose-dependent manner (Figures 4G–M). These results demonstrated that ATMP reduces glycogenesis by suppressing PI3K/Akt/GSK3β signaling pathway (Figure 4A).

### 3.8. *Acanthopanax trifoliatus* (L.) Merr polysaccharide reduces hepatic glycogenolysis by inhibiting the cAMP/PKA signaling pathway in T2DM mice

**Figure 5:**
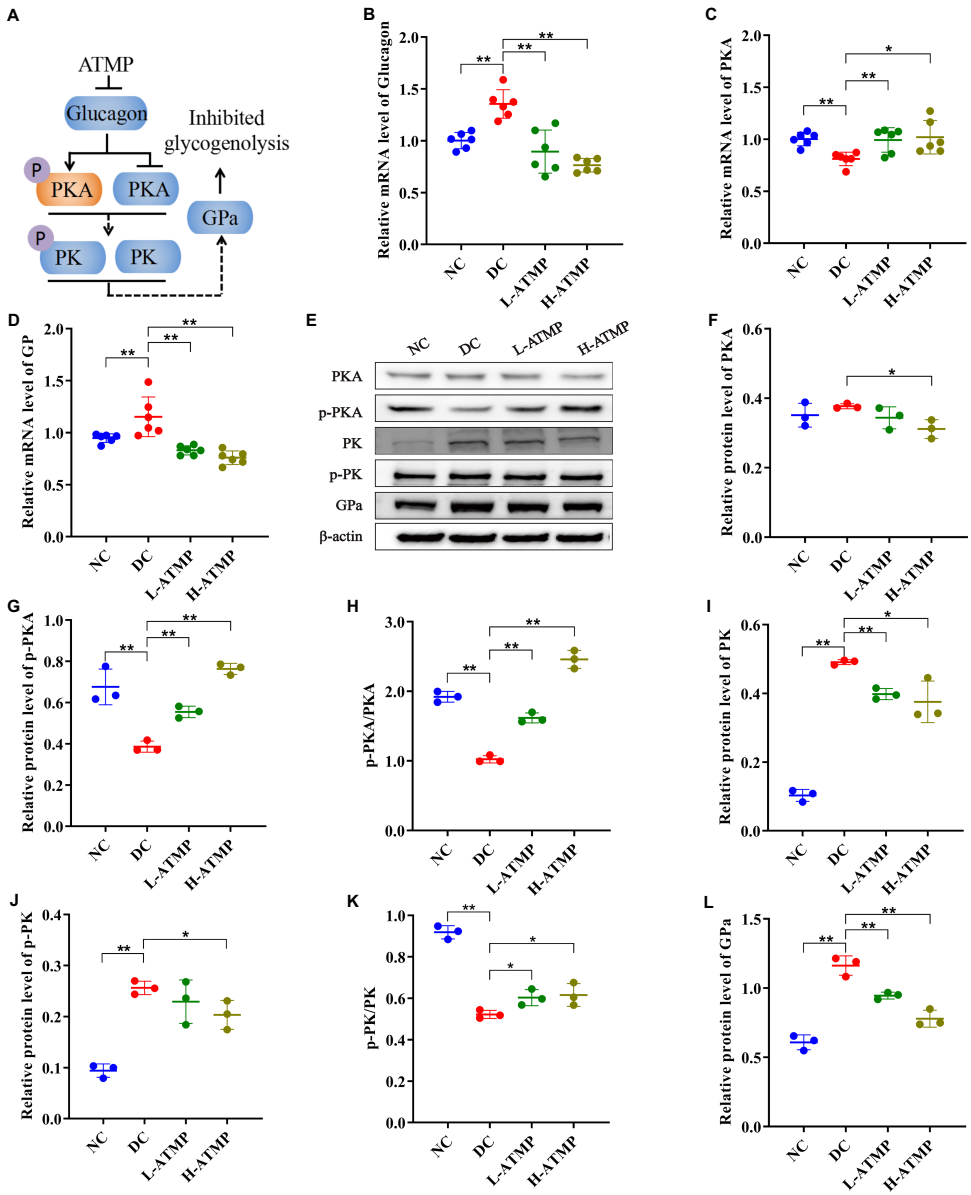
*Acanthopanax trifoliatus* (L.) Merr polysaccharide (ATMP) inhibits hepatic glycogenolysis *via* the cAMP/PKA signaling pathway. The T2DM mice were administered 40 mg/kg or 80 mg/kg ATMP by oral gavage for 8 weeks, then the mRNA and protein levels of cAMP/PKA signaling pathway-related proteins were determined. **(A)** Schematic diagram showing ATMP mediating cAMP/PKA signaling to suppress hepatic glycogen synthesis. Orange nodes: up-regulated proteins; Blue nodes: down-regulated proteins. **(B–D)** Relative mRNA levels of Glucagon, PKA and GP, and β-actin as a loading control were assayed by RT-qPCR. The relative mRNA levels for proteins of interest were normalized to β-actin. **(E)** Relative protein levels of PKA, p-PKA, PK, p-PK and GPa, and β-actin as a loading control were assayed by Western blot. Quantifications of band intensity were normalized to β-actin **(F–L)**. Results are expressed as the mean ± SD. ^*^p < 0.05 and ^**^p < 0.01 vs. DC. NC: normal group; DC: diabetic control group; L-ATMP and H-ATMP: diabetic mice treated with ATMP at 40 or 80 mg/kg, respectively.

The cAMP/PKA signaling pathway regulates glucose homeostasis at multiple levels (38). GP is the rate-limiting enzyme of glycogenolysis, and GPa is its active form (39). In the process of liver glycogenolysis, cyclic adenosine monophosphate (cAMP) is elicited by the binding of glucagon to GPCR, which activates cAMP-dependent protein kinase (PKA) to stimulate the expression of GPa and then promotes glycogenolysis (40). To elucidate the hypoglycemic mechanism of ATMP in mediating glycogenolysis, the mRNA levels of key factors that participated in the cAMP/PKA signaling pathway were examined by RT-qPCR in all groups. We found that ATMP up-regulated the expression of PKA and down-regulated the expressions of glucagon and GPa (Figures 5B–D). In addition, the levels of key proteins in the cAMP/PKA signaling pathway were measured by western blot. Compared with untreated diabetic mice, we found that ATMP treatment induced a marked increase of p-PKA and p-PKA/PKA, and a decrease of PKA, PK, p-PK, GPa, resulting in an inhibition of liver glycogen breakdown (Figures 5E–L). These results elucidated that the cAMP/PKA signaling pathway was involved in the ATMP-mediated inhibition of glycogenolysis (Figure 5A).

### 3.9. *Acanthopanax trifoliatus* (L.) Merr polysaccharide suppresses hepatic gluconeogenesis by activating the AMPK signaling pathway in T2DM mice

**Figure 6:**
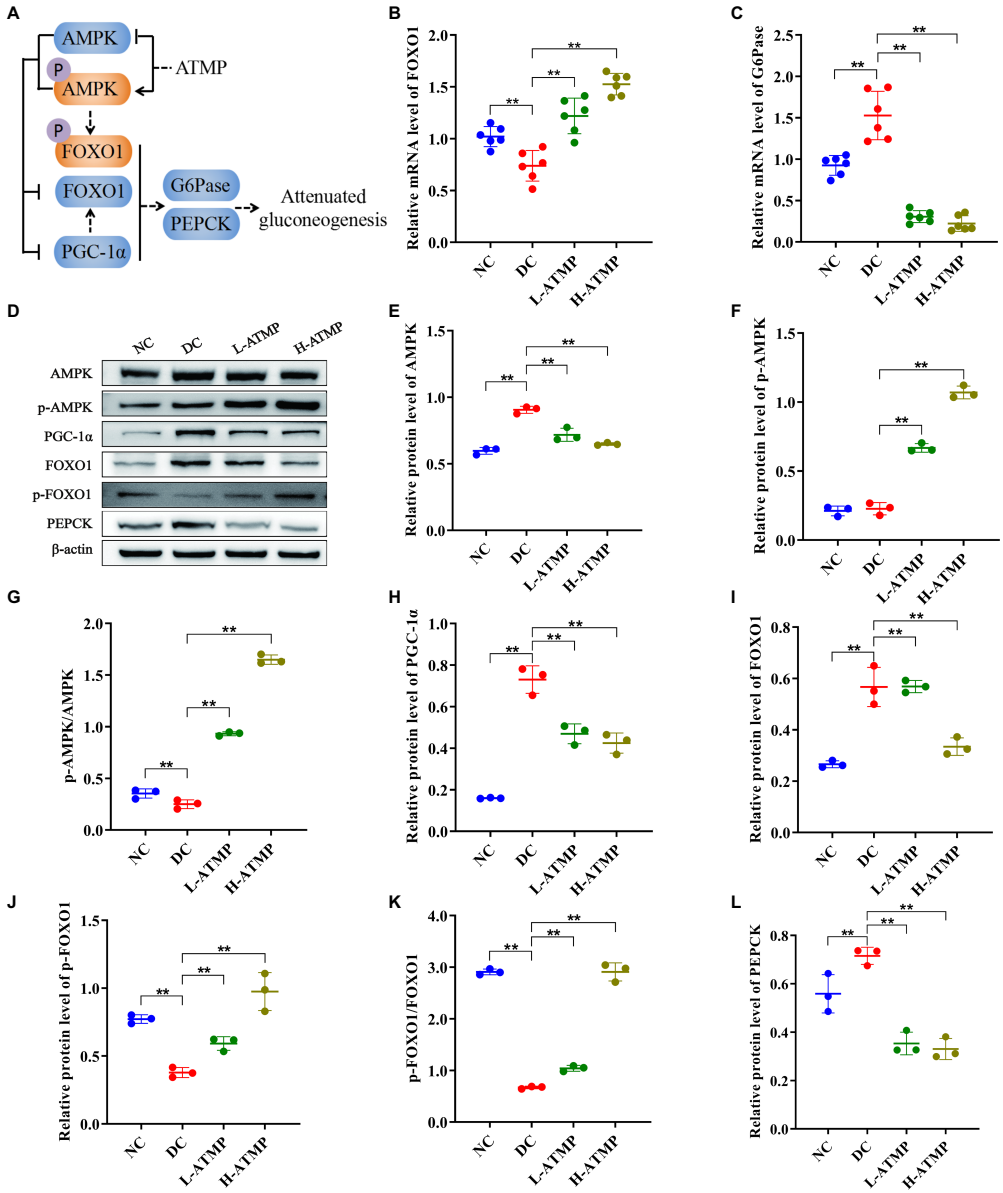
*Acanthopanax trifoliatus* (L.) Merr polysaccharide (ATMP) suppresses hepatic gluconeogenesis *via* the AMPK signaling pathway. The T2DM mice were administered 40 or 80 mg/kg ATMP by oral gavage for 8 weeks, then the mRNA and protein levels of AMPK signaling pathway-related proteins were determined. **(A)** Schematic diagram showing ATMP mediating AMPK signaling to suppress hepatic gluconeogenesis. Orange nodes: up-regulated proteins; Blue nodes: down-regulated proteins. **(B–C)** Relative mRNA levels of FOXO1 and G6Pase, and β-actin as a loading control were assayed by RT-qPCR. The relative mRNA levels for proteins of interest were normalized to β-actin. **(D)** Relative protein levels of AMPK, p-AMPK, PGC-1α, FOXO1, p-FOXO1 and PEPCK, and β-actin as a loading control were assayed by Western blot. Quantifications of band intensity were normalized to β-actin **(E–L)**. Results are expressed as the mean ± SD. ^*^p < 0.05 and ^**^p < 0.01 vs. DC. NC: normal group; DC: diabetic control group; L-ATMP and H-ATMP: diabetic mice treated with 40 or 80 mg/kg ATMP, respectively.

The liver is the major organ for gluconeogenesis. PEPCK and G6Pase are two crucial rate-limiting enzymes in the gluconeogenesis pathway, and their transcription levels of them determine the rate of gluconeogenesis (41). Previous studies have demonstrated that inhibiting FOXO1 reduced the expression of PEPCK and G6Pase (42). And the activity of FOXO1 is regulated by AMPK, suggesting that the AMPK pathway plays an important role in gluconeogenesis (43, 44). To further elucidate the mechanism of ATMP in mediating gluconeogenesis, we evaluated the mRNA and protein levels of molecules related to the AMPK signaling pathway in the liver. ATMP induced the transcription of FOXO1 and inhibited the transcription of G6Pase in the liver (Figures 6B,C). Compared to the diabetic control group, the hepatic protein levels of AMPK, FOXO1, PGC-1, and PEPCK were significantly down-regulated in the ATMP groups. In contrast, the hepatic protein expression levels of p-AMPK, p-AMPK/AMPK, p-FOXO1, and p-FOXO1/FOXO1 were significantly up-regulated after ATMP treatment (Figures 6D–L). These data indicated that ATMP could inhibit hepatic gluconeogenesis by activating the AMPK signaling pathway (Figure 6A).

## 4. Discussion

**Figure 7:**
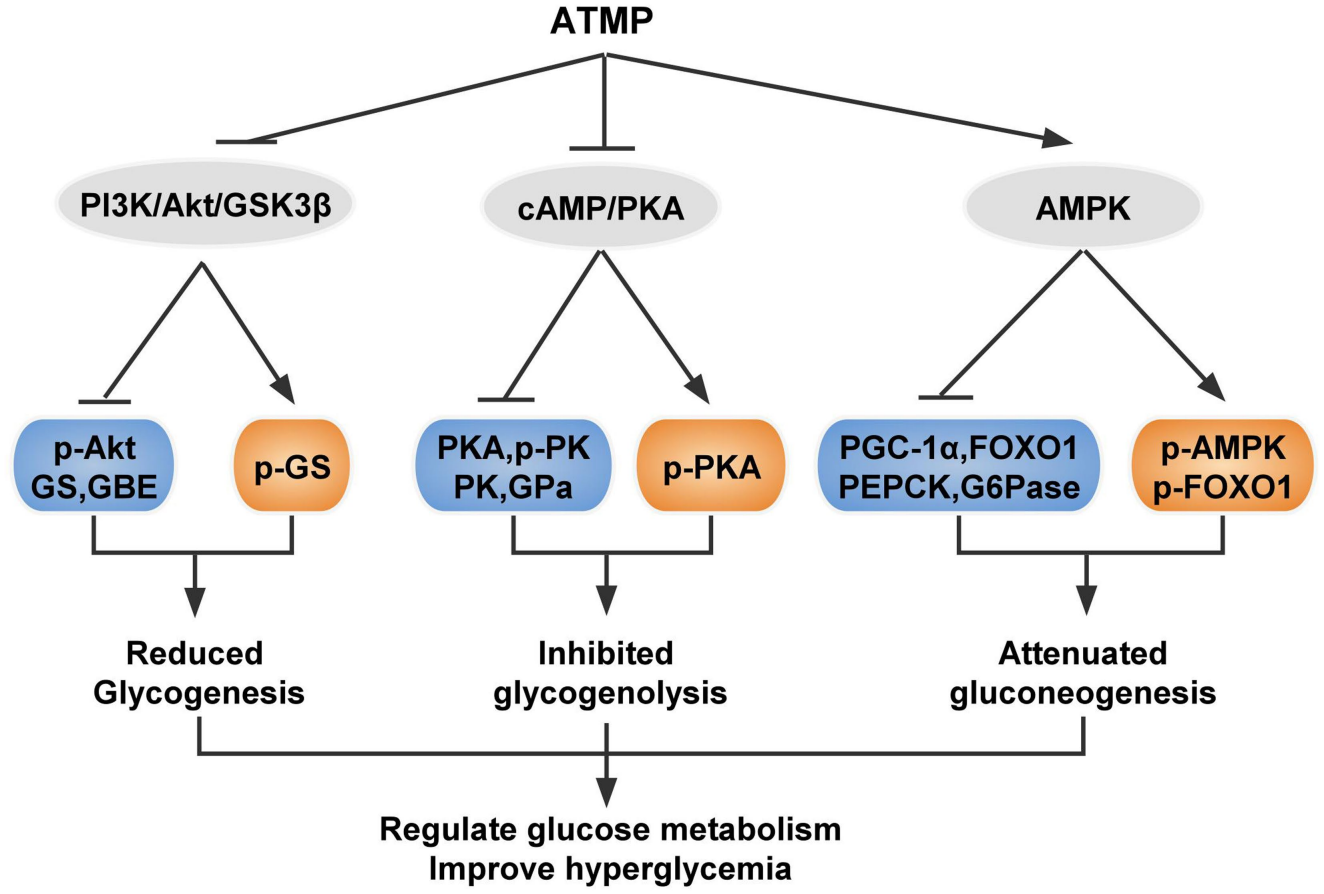
Schematic diagram showing the proposed mechanism of ATMP ameliorating hyperglycemia in T2DM mice. Orange nodes: up-regulated proteins; Blue nodes: down-regulated proteins.

Developing novel drugs which have fewer side effects without loss of insulin sensitization is critical for improved therapies for T2DM. Here, we found that ATMP, a bio-macromolecule isolated from *Acanthopanax trifoliatus* (L.) Merr showed effective hypoglycemic ability against T2DM. Our studies demonstrated that ATMP possessed anti-hyperglycemic properties and the mechanism is relevant to glycogen metabolism. ATMP could reduce glycogenesis *via* PI3K/Akt/GSK3β signaling pathway, suppress glycogenolysis by inhibiting cAMP/PKA signaling, and attenuate gluconeogenesis by activating the AMPK signaling pathway (Figure 7). Therefore, ATMP is a potential therapeutic agent for the treatment of T2DM.

Glucose homeostasis is critical to human health. Multiple layers of regulation are required for maintaining it and various pathways of glucose metabolism are involved, including glycogenesis, glycogenolysis, glycolysis and gluconeogenesis (25). Dysregulation of glucose homeostasis may lead to hyperglycemia associated conditions such as obesity and type 2 diabetes mellitus. Many anti-diabetic drugs exert clinical effects *via* different mechanisms, including biguanides, insulin secretagogues, insulin sensitizers, and insulin or its analogs (45). The major classes of oral antidiabetic medications include biguanides, sulfonylureas, meglitinide, thiazolidinedione (TZD), dipeptidyl peptidase 4 (DPP-4) inhibitors, sodium-glucose cotransporter (SGLT2) inhibitors, and α-glucosidase inhibitors (46). Although plenty of diabetic drugs exist, drug monotherapy proved unsuccessful in providing satisfactory management of blood glucose levels and other comorbidities. Therefore, therapeutic management is usually achieved by combining therapy with drugs that act with different mechanisms. For instance, a combination therapy of vildagliptin plus metformin provides greater and durable long-term benefits than metformin monotherapy for patients with newly diagnosed type 2 diabetes (47). However, the combination strategy may be affected by problems related to polypharmacological drug actions, such as undesirable side effects, toxicity, and unwanted drug–drug interactions (48). Sulfonylureas and insulin are associated with an increased hypoglycemia risk and would not be preferred for patients in whom this is a concern (49). To solve the problem caused by combination therapy, a single molecule that selectively modulates multiple targets was considered a new therapeutic strategy for T2DM.

The action mode of multi-targets for bioactive compounds isolated from natural plants could be a promising treatment of diabetes mellitus. In a previous research programme, 29 hypoglycemic components and 63 hypoglycemic targets of *Polygonum multiflorum* (PM) were filtrated. And 38 metabolic pathways were connected with the hypoglycemic mechanism of PM (50). Polysaccharides are one of the main components of the natural sources and have been applied for the diabetes mellitus treatment during recent years. Tea polysaccharides were found to increase the body weight and decrease the blood glucose. Polysaccharides of corn silk were reported for its antidiabetic effects on diabetic rats. Polysaccharides from *Lachnum calyculiforme* and polysaccharides from *Cynomorium songaricum* were proved to exhibit obvious hypoglycemic effects (51). Polysaccharides extracted from natural products might be a promising candidate for T2DM treatment by targeting different signaling pathways. In our study, we evaluated the antidiabetic effects of polysaccharides isolated from *Acanthopanax trifoliatus* (L.) Merr. The results indicated that ATMP exhibited noticeable anti-hyperglycemic activity.

*Acanthopanax trifoliatus* (L.) Merr is a drug-food homologous plant and has been reported to have various health benefits including diabetes treatment. For example, the roots and bark of *Acanthopanax trifoliatus* (L.) Merr is effective in improving rheumatism and diabetes (52). Polysaccharides isolated from *Acanthopanax trifoliatus* (L.) Merr can reduce blood glucose levels by restoring the immune balance in the spleen (14). In this study, we demonstrated that ATMP is a multi-targets ligand and plays a hypoglycemic role through synergistic and multifactorial approaches. ATMP affected glycogen metabolism *via* regulating PI3K/Akt/GSK3β, cAMP/PKA, and AMPK signaling. The results showed that ATMP exhibited potent anti-hyperglycemic activity, being as efficacious as Metformin. In addition, ATMP did not seem to have any toxic effects on mice. Compared with traditional “one gene, one drug, one disease” mode, polypharmacology, which focuses on multi-target drugs, has emerged as a new paradigm in drug discovery. Strategies based on network may play a useful role in antidiabetic drug discovery (51). We are currently examining the effect of ATMP through a network pharmacology approach. With the rapid progress of bioinformatics and integrating network biology, we believe that the appropriate use of network pharmacology approaches may initiate new directions and contribute leading insights into the discovery of novel antidiabetic drugs.

## 5. Conclusion

In conclusion, ATMP could promote insulin secretion and inhibit glucagon secretion, effectively improve glucose tolerance and alleviate insulin resistance. ATMP decreases glycogen synthesis, reduces glycogenolysis, and inhibits liver gluconeogenesis in T2DM mice, repairing liver impairment by regulating glucose metabolism. ATMP is a promising therapeutic component for treating type 2 diabetes.

## Data availability statement

The original contributions presented in the study are included in the article/supplementary material, further inquiries can be directed to the corresponding authors.

## Ethics statement

The animal study was reviewed and approved by The Ethics Committee of the experimental animal center of Guangdong Pharmaceutical University.

## Author contributions

YP, GW, and HY: conceptualization, supporting, funding, and supervision. YzL, JP, and YL: conducting experiment and data analyses. YzL and YL: writing original draft preparation. YP and GW: review and editing. All authors contributed to the article and approved the submitted version.

## Funding

This research was funded by the Natural Science Foundation of Guangdong Province (2017A030313623). This study was also supported by Medical Scientific Research Foundation of Guangdong Province of China (A2022182). This study was also supported by Scientific Research Funding of the First Affiliated Hospital of Guangdong Pharmaceutical University (KYQDJF202016). This study was also supported by National Key Clinical Specialty Construction Project (Clinical Pharmacy) and High Level Clinical Key Specialty (Clinical Pharmacy) in Guangdong Province.

## Conflict of interest

The authors declare that the research was conducted in the absence of any commercial or financial relationships that could be construed as a potential conflict of interest.

## Publisher’s note

All claims expressed in this article are solely those of the authors and do not necessarily represent those of their affiliated organizations, or those of the publisher, the editors and the reviewers. Any product that may be evaluated in this article, or claim that may be made by its manufacturer, is not guaranteed or endorsed by the publisher.
